# Changes in bioelectrical and non-bioelectrical variables associated with overweight after a weight-loss program based on intestinal microbiota modulation

**DOI:** 10.2478/joeb-2025-0018

**Published:** 2025-12-17

**Authors:** L. O. Tapasco-Tapasco, C. A. Gonzalez-Correa, P. A. Gomez-Buitrago

**Affiliations:** Research Group on Bioelectrical Impedance (GruBIE), University of Caldas (UC), Manizales, Colombia, South America; Department of Chemistry, University of Cauca, Popayán, Colombia, South America

**Keywords:** Bioelectrical impedance analysis, overweight, dyslipidemia, hydrotherapy, gut microbiota modulation, weight loss

## Abstract

**Objective:**

To assess changes in clinical and bioelectrical impedance analysis (BIA) variables associated with overweight, after a 6-day weight-loss protocol based on intestinal microbiota modulation.

**Methods:**

A group of 12 young overweight women (OG) were randomly assigned to either a control subgroup (COG, *n*=6) or an experimental subgroup (EOG, *n*=6), while, for comparison, eight lean healthy women served as reference (LG). The intervention combined a liquid diet, probiotics, psyllium, bentonite, and a daily open-system trans-anal irrigation. 23 clinical variables not involving BIA (type BIA-0: 12 physical, 9 chemical and 2 biological), and 21 variables obtained by BIA were measured at 4 time points (T1-T4), in a time lapse of 10 weeks. 11 BIA variables were designed as BIA-1, i.e., proper bioimpedance variables, and 10 as BIA-2, i.e., those calculated by a combination of BIA-0 and BIA-1 variables. Intestinal microbiota (IMB) modulation was explored via two biological variables: *Firmicutes*/*Bacteroidota* ratio and *Akkermansia muciniphila* relative prevalence. All variables (except age and height) were also divided in two subtypes: “+”, those usually higher in overweight people, whose median values were expected to decrease after the intervention (a total of 26), and “−”, those usually lower in overweight people, whose medians values were expected to increase with the intervention (a total of 16).

**Results:**

all 42 variables susceptible to changes with the intervention changed in a favorable direction (their median values moved towards those of the LG), with 32 of the changes showing statistical significance.

**Conclusions:**

In this pilot study, a multimodal microbiota-oriented protocol was associated with consistent and, mostly, clinically meaningful improvements of bioelectrical and physiological markers in overweight young women. Changes in BIA parameters seem to mirror the physiological changes detected in BIA-0 variables. Larger and longer trials are warranted to confirm these findings.

## Introduction

The most common biomedical use of electrical bioimpedance is for the study of body composition and, for this purpose, the technique is known as electrical bioimpedance analysis (BIA) [1]. Hyper adiposity (overweight/obesity) is a medical condition that, like most chronic non-communicable diseases (CNCDs), undergoes a common pathway that can be named as the pathophysiological cascade of chronicopathy, a term we use to name the condition of suffering any CNCD [2]. Thus, chronicopathy begins with unhealthy life habits (four of which are commonly mentioned: sedentarism, unhealthy diet, tobacco use and excessive alcohol consumption [3]), translating into dysbiosis of the intestinal microbiota (IMB), metainflammation and then, in overweight, with alteration of body composition, especially in terms of increased body fat mass and, in many cases, decreased muscle mass and function. Conventional leaning programs are mainly based on improving lifestyle: diet, physical activity and behavior [4] but, more recently, modulation of IMB composition has also been considered [5, 6], even by means of fecal transplantation [7], a term that has been included in the PubMed thesaurus since 2016 (MeSH, Medical Subject Headings [8]).

**Figure 1:**
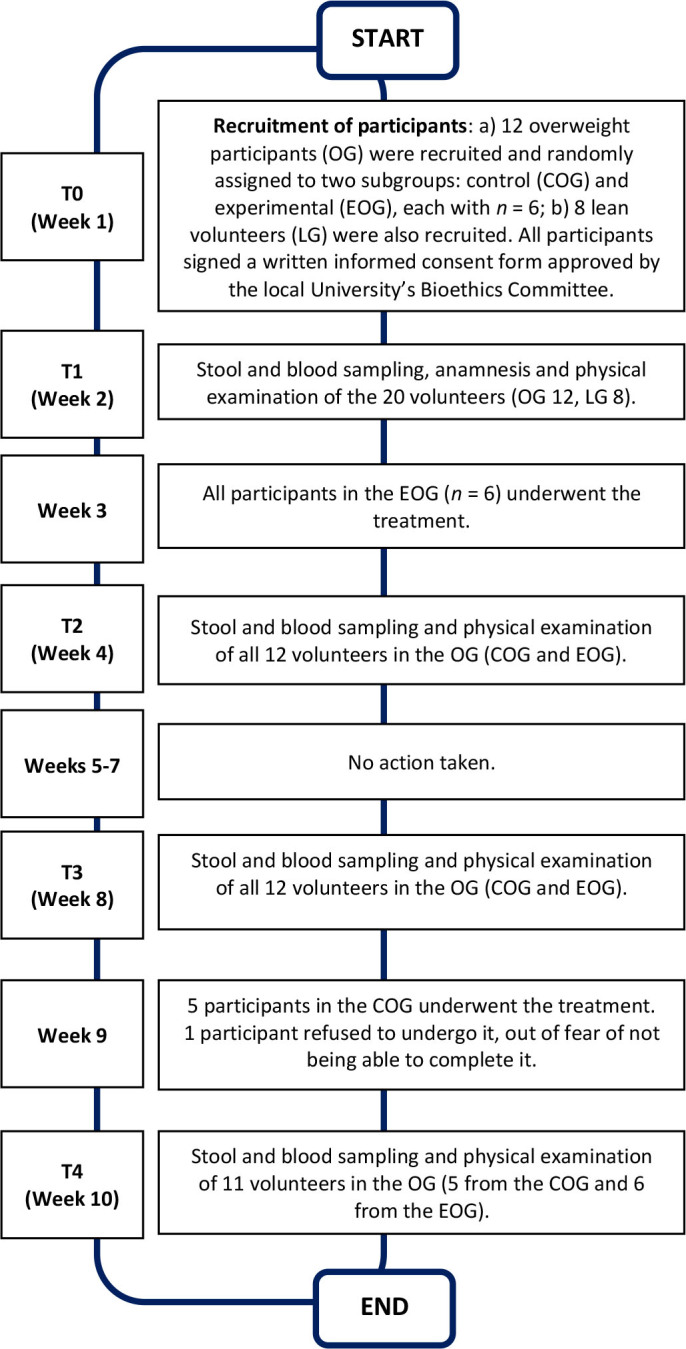
Timetable of the study. T1-T4 indicate the four times when samples and measurements were taken in the OG.

In this study, which, to some extent, can be considered as a pilot descriptive-exploratory one, 11 individuals of a group of 12 overweight, female, young adults (OG) with dyslipidemia underwent a 6-day weight loss intervention through modulation of the IMB, an intervention whose principles and main characteristics are presented by González-Correa *et al.* 2017 and Tapasco-Tapasco *et al.* 2024 [2, 9]. An additional group of 8 lean, female individuals (LG) of the same age group and with not known medical conditions was also recruited, to compare not only the post-intervention results with the pre-intervention ones, in the OG, but also with the values of a group of assumed lean, healthy people (LG) of the same gender and age, which, for this study, are taken as reference values. At the beginning of the study, the OG was randomly divided into two subgroups (see **Figure 1**): one named control overweight group (COG) and another one named experimental overweight group (EOG), each with an *n* = 6. All six participants in the EOG had the intervention at the beginning of the study, and 5 of the COG towards the end of it (one individual of this group declined the intervention, and her data were not included in the analysis).

Three types of physiological variables (44 in total), mostly associated with overweight/obesity, were measured, which, in this article, will be called BIA-0, BIA-1 and BIA-2: variables not directly related to BIA (BIA-0, *n*=23), primary BIA variables (BIA-1, *n*=11, those actually measured by the technique, i.e. those directly related to the electrical impedance or its modelling), and secondary BIA variables (BIA-2, *n*=10, those calculated using BIA-1 measurements, combined with some of the BIA-0 variables). In the OG, measurements were taken at four different times (T1-T4), as specified in **Figure 1**, and the study lasted for 10 weeks.

All variables were also classified in two subtypes: 1) those whose values, in overweight/obese people, are usually elevated and, therefore, their decrease can be considered as beneficial, as, for instance, weight, percentage body fat, and low density lipoprotein (LDL) (subtype “+”, *n*=26) and which, in principle, ought to decrease with the treatment, and 2) those whose values, on the contrary, are usually diminished in overweight/obese people and, therefore, ought to increase with the treatment (subtype “−”, *n*=16), as, for instance, muscle mass and function, and high density lipoprotein (HDL). Age and height (type BIA-0) are two variables that do not change and, therefore, are not included in either subtype.

Medians of the variables in the OG were compared before and after the intervention, and, in both cases, against the medians of the variables in the LG. As indicators of IMB modulation, two variables were measured: 1) the *Firmicutes* to *Bacteroidota* ratio (F/BR), which some authors consider as an indicator of adiposity [10], and 2) the relative prevalence of the bacterial species *Akkermansia muciniphila* (%AM), which has been inversely associated with hyper adiposity [11].

The main aim of the study was twofold: a) on one side, to see the effect of the IMB modulation in the selected BIA-0 variables, and b) to see if BIA related variables (BIA-1 and BIA-2) mirrored those changes.

## Materials and methods

### Subjects

By convenience sampling, volunteers participating in the study were recruited from local universities’ students, and the treatment was carried out during the COVID-19 pandemic. All of them signed an informed consent form approved by the Bioethics Committee of the Faculty of Health Sciences (CBCS) at the local university where the study took place (protocol approved on 28-05-2019, communication CBCS-043). Initially, 12 overweight, young, dyslipidemic, adult, female volunteers with dyslipidemia were recruited and accepted to participate in the OG. A second group of 8 healthy, lean, young, adult, female volunteers was also recruited for the LG, had all variables measured only once and was not treated.

### Inclusion criteria

- Being female aged > 18 years and < 26 years, verified by an identity document.
- Body mass index (BMI) > 25.0 and < 30.0 kg/m^2^ for the OG and BMI > 18.5 and < 25.0 kg/m^2^ for the LG.
- Percentage body fat (%BF) > 30.0% of total body weight, established by BIA, for the OG and < 30.0 % for the LG.
- Having no comorbidities or other contraindications, such as those mentioned in the exclusion criteria, established by anamnesis, for the OG group, and not having any known medical condition for the LG, also established by anamnesis.
- Willing to undergo the intervention, for the OG group.
- Signing informed consent in the presence of a witness and one of the researchers involved in the study.

### Exclusion criteria

- Obesity, diabetes or hypothyroidism.
- Severe comorbidities considered as contraindications for colonic cleansing: epilepsy, severe heart disease, severe anemia, hepatic cirrhosis, renal insufficiency, abdominal hernia, recent colon surgery, cancer, necrosis from abdominal irradiation, acute hemorrhagic colitis, suspicion of intestinal perforation, advanced anal fissures/fistulas, advanced hemorrhoids or rectal bleeding.
- Recent use of medications (within the previous 6 months) for weight reduction, modulation of lipemia or treatment of hypertension.
- Previous bariatric surgery.
- Considerable weight change prior to starting the study (within the last 6 months).
- Alcoholism or any type of pharmacological addiction.
- History or suspicion of familial dyslipidemia.
- Being pregnant or lactating or intending to become pregnant soon.
- Psychiatric illness or other chronic illness.
- Use of antibiotics within the 3 months prior to the intervention.
- Not willing to follow the designed study protocol.
- Not willing to sign the informed consent form.

All these criteria were assessed by anamnesis.

### Variables included in the study

The complete list of the 44 variables measured in the study is presented as Supplementary Information (SI) in **Table SI-1**, where their units are also included, as well as the medians of the values obtained, both for the single measurement of the LG and pre- and post-treatment, for the OG. Also included are the *p*-values for the comparison of pre- and post-values, and the significance of the changes. For BIA measurements, a BiodyXpert^ZM^ from Aminogram (France) was used. This is a tetrapolar device which operates with no external electrodes, i.e., all four electrodes are directly attached to the device, where two of them make contact with the fingers of the right hand and another two with the inframalleolar ipsilateral area [12]. (**Note**: for data analysis, 7 of the BIA-2 variables given in either kg or L by the device, were converted into percentages of body mass, i.e., body weight). The genomic sequencing of the bacterial DNA obtained from the stool samples was carried out at the biotechnology laboratory Corpogen [13], using Illumina technology, after PCR amplification of regions V3 and V4 of the 16S rRNA gene.

### Statistical analysis and presentation of the data

Results for the analysis are all continuous quantitative variables, which were reported with one decimal place and presented as medians. For the comparison of values between groups and those obtained pre- and post-treatment, a Student’s *t*-test was used for data with a normal distribution, while a Wilcoxon-Mann-Whitney test was used for data with a non-normal distribution. Normality was assessed by the Shapiro-Wilk test. Tests calculations were performed using the statistical program SPSS version 22.0. For simplicity, the data of all variables at the four measurement times (T1-T4, see **Figure 1**) were normalized towards the values of the LG, where the median values of this group are considered as 100%.

### Experimental protocol

The main products used during the 6-day treatment are listed in **Table SI-2**, specifying their three main macronutrients (protein, carbohydrates and fat), daily intake and number of calories. **Figure 1** shows the timetable of the study.

More details on the protocol can be obtained in González-Correa *et al.* 2017 and Tapasco-Tapasco *et al.* 2024 [2, 9]. Basically, it consists of a 6-day protocol, where people eat a primarily vegetarian, liquid diet, consume pre- and probiotics and undergo a daily trans anal irrigation during the first 5 days, the latter carried out using a Colema^®^ Board [14]. Two natural products, psyllium and bentonite, were also consumed daily.

As mentioned before, all participants in the EOG underwent the intervention at the beginning of the study (week 3) and 5 of the COG did it at the end of it (week 9). This was designed, firstly, to see if the changes obtained immediately after the intervention persisted at least one month later and, secondly, to have a greater sample of treated individuals (12 instead of 6, although, as already mentioned, one volunteer of the COG declined to undergo the 6-day intervention, out of fear of not being able to complete it and, therefore, her data were not included in the analysis).

### Ethical approval

The research complied with all relevant national regulations related to human participants and institutional policies, was carried out in accordance with the tenets of the Helsinki Declaration and had been approved by the Bioethics Committee of the Faculty of Health Sciences at the local university where the study was carried out (Consecutive number CBCS-043).

### Informed consent

Informed consent was obtained from all individuals included in this study.

## Results

**Figure 2:**
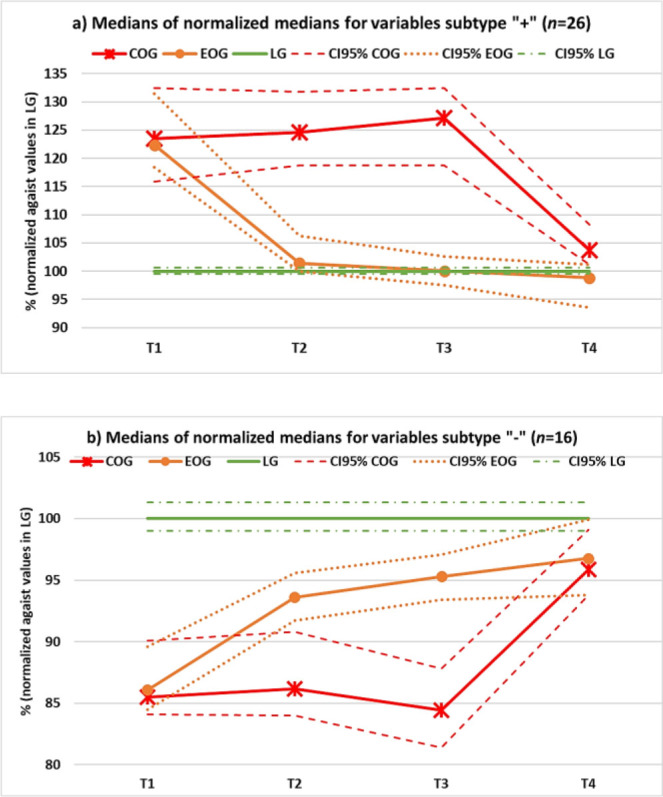
Behavior, during the study, of medians of the variables pertaining to: a) subtype “+”, i.e., those whose decrease can be considered as beneficial, and b) subtype “−”, i.e., those whose increase can be considered as beneficial. T1 to T4 refer to the time of measurement, and values in parenthesis indicate the number of variables (n) included in each type. CI95%: indicate 95% confidence intervals. For more details see text and Supplementary Information.

All medians of the 42 variables susceptible to changes associated with the intervention included in this study did change, in the OG, in what can be considered a beneficial direction, after treatment (“post-” values), when compared to the median values before it (“pre-” values). Medians of the raw data for the pre and post values in the OG, as well as those of the LG, are given as Supplementary information in **Table SI-1**. **Figure 2** shows the behavior of the medians of the two subtypes of variables (a) “+” and b) “−”, for the two subgroups of the OG (COG and EOG), where the data corresponding to the single measurements in the LG is depicted as a straight line, taken as reference and, therefore, with a value of 100%.

**Figure 3:**
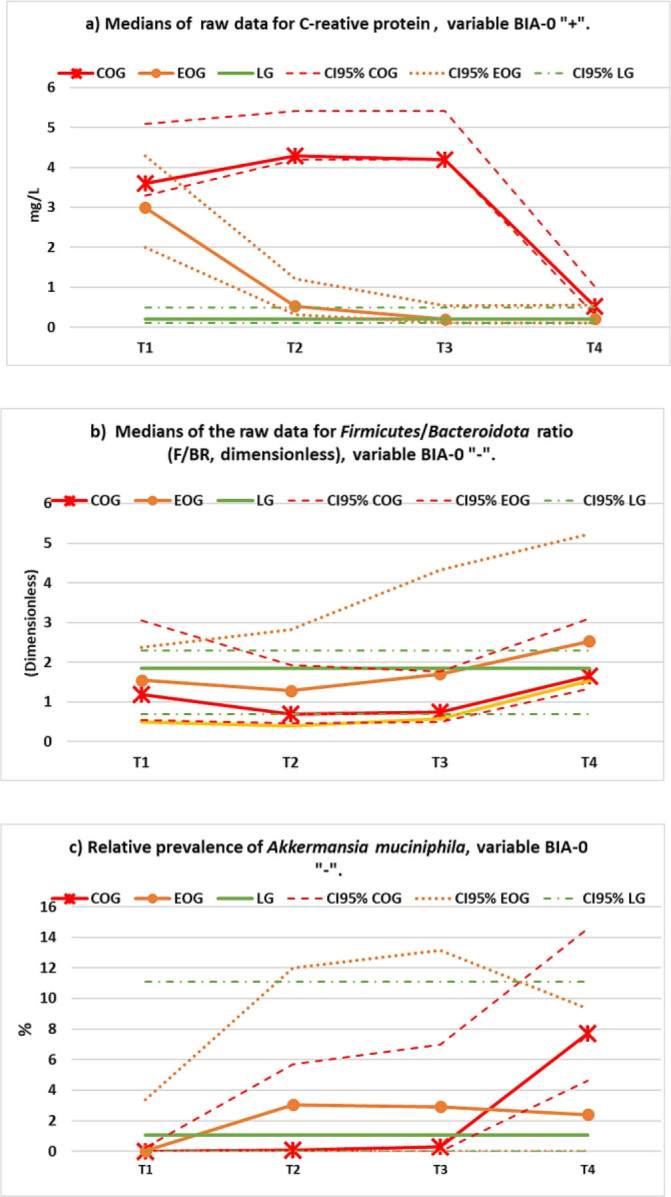
Three special cases of variables type BIA-0: a) CRP (a subtype “+” variable), b) F/BR, and c) %AM, the latter two, subtype “−”. See text for more details.

Three special cases are worth highlighting: CRP, F/BR and %AM, not only for their significance for the study, but also because they present the highest per cent changes between post- and pre-intervention measurements, as shown in **Table SI-1** (−87.62%, +64.86% and +6197.81%, respectively). CRP, an indicator of metainflammation, is a very special case given that the median value for that variable, in the LG, taken as reference for the normalization, was very low and, therefore, the difference of the initial median values in the OG (i.e. COG and EOG), when compared to the median of the LG, is very large (about 21-fold). The behavior of this variable is the same as the behavior of the other variables of its type, as can be seen in **Figure 3 a)**. The F/BR is also special, because many articles suggest that this variable is higher in overweight and obese people, although the results of other studies show the contrary. In this case, the median value of the F/BR is lower in the OG, when compared to the median of the LG, at the beginning of the study, and, after treatment, it increases substantially, surpassing the value of that of the LG, but, apparently, with a delay in the EOG participants, as shown in **Figure 3 b)**. Despite its large per cent change, though, the difference between pre- and post-values is not statistically significant, as indicated in **Table SI-1**. Finally, the median of the relative prevalence %AM is very interesting because this bacterial species, in the OG, goes from a median of almost zero, at the beginning of the study, to 5.5 times the median of the LG, at the end of the study (see **Figure 3 c**, and **Table SI-1**).

For variables type BIA-1, those considered to be related to metainflammation show a similar behavior to those from type BIA-0: a) the median value of the impedance ratio (IR or Z200/Z5, a subtype “+” variable) went down, and b) those of phase angle (PhA) and reactance at 50 kHz (Xc50), two subtype “−” variables, went up. Despite this, it must be said that, while changes in IR and PhA are statistically significant, the 15.7% increase in the median of Xc50 is not.

Finally, it also has to be mentioned that, apart from PhA and IR, the changes in the median values of the rest of the BIA-1 variables (R and Z at the different frequencies used by the device) are not statistically significant, although they moved, after treatment, in a direction in concordance with the changes in body composition, i.e., the values became lower as water and muscle mass increased, and fat mass decreased. Changes in the BIA-2 variables also moved in a favorable direction in the OG, after treatment, and, in this case, in all but one (metabolic protein mass) of them (out of 11), the changes were statistically significant.

## Discussion

From the initial group of 12 volunteers recruited for the OG, one decided to withdraw when she came to undergo the treatment, arguing fear of not being able to complete the 6-day intervention, especially because habitual food is excluded from it (see **Table SI-2**). Consequently, the data of this participant were excluded from the analysis.

From the results shown, the first and most important finding to highlight is that the 42 variables included in this study, susceptible to treatment-associated changes, moved in what can be considered as a favorable or beneficial direction, i.e., towards the median values of the variables measured in the LG group, which were taken as reference. The majority of changes (31) were statistically significant, very notorious and, some of them, even dramatic, as, for instance those already mentioned of CRP, F/BR and %AM. Interestingly, the changes in the post- versus pre-treatment values in both BIA-0 and BIA-2 variables, in each group, all them, but one in both cases, were statistically significant, while, in the BIA-1, the opposite was true, in that the changes of only 2 out of 11 variables were statistically significant (PhA and Xc50).

In relation to the median weight loss after treatment, (−3.4 kg), it is interesting to note that this represents a decrease of almost 5.0% (4.9%), which, by some authors, is taken as the minimum percentage decrease to consider a leaning treatment as successful [15, 16], especially if maintained with time. In this study, in the EOG, the median pre-treatment weight (data not shown) went from 69.9 kg to 66.3 kg (at times T1 and T2, respectively), and, at least two months later, without any further intervention, the median for this group was still 66.2 kg (data not shown).

In relation to the lipid profile, although it was difficult to find individuals of the target population (college, female, young, overweight adults) with overt dyslipidemia, all participants had at least 1 of the profile’s variables outside the reference values (data not shown), especially high-density lipoprotein (HDL, all of them), and triglycerides (TG, 6 out of 11). Nevertheless, it is worth pointing to the dramatic decrease in the latter variable, showing a reduction in its median value, for the OG, after treatment, to less than half its initial value (it went from 177.0 mg/L to 79.0 mg/L, a reduction of 55.4% of its initial value, as shown in **Table SI-1**), and bringing it well within the general reference value of <170 mg/dL. There are two other interesting findings in relation to this profile: a) the fact that, while “bad” cholesterol decreased, the “good” cholesterol increased, simultaneously, and b) the improvement in the TG/HDL median value in the OG which went from “good” (3.6) to “very good” (2.6), with a statistically significant change of −28.1%.

In relation to the LG, it only had three variables with medians outside the reference range: Tecumseh test cardiac rate (85.0 beats/min), HDL (54.5 mg/dL) and F/BR (1.9). However, of these, the latter two medians were relatively close to the normal range. For the F/BR, it is a special case, as, although, many authors consider that it has a negative correlation to being overweight/obese there is some controversy in relation to this assertion, as some authors have shown an opposite correlation [9]. Interesting, though, is the fact that, while the median of this variable, for the OG before treatment (1.2), showed a far lower value than that of the LG (1.6) and closer to what Koliada *et al.* 2017 [15] consider as the ceiling of their reference range (1.0), the main value of the median for the OG group, after treatment, moved closer to that of the LG, taken as an intra-study reference. Although the pre- and post-treatment values of this variable are not statistically different, the median increase is, still, very high, as shown in **Table SI-1** (64.9%).

In relation to the biomarkers of metainflammation, with data of this same study, a very good correlation was found between the biochemical indicator of this condition (CRP, a BIA-0 variable) with two of the BIA-1 variables (IR, subtype “+” and PhA, subtype “−”) [8]. Finally, concerning BIA related variables, we would like to mention some findings of the data given by the device used in this study:

1. One aspect that attracted the authors’ attention, was the fact that the values for PhA were very high in all measurements, well above the accepted general reference values.
2. In order to test consistency, with the data provided by the same device, PhA was calculated in two ways (data not shown): as arcsine (Xc50/Z50) and as arctangent (Xc50/R50), giving exactly the same values in both cases, but differing from the values presented by the equipment, with relative differences going from −18.4% up to 34.2%, with mean of −9.6% and standard deviation (SD) of ±5.2% for the negative differences, and a mean of 9.5% and SD of ±8.7% for the positive differences.
3. “Fat free hydration” is different from “hydration of the fat free mass”, as the former removes the water contained in adipose tissue, while the latter does not. When dividing total body water by fat free mass (lean mass), the values calculated in the 52 measurements gave a mean of 0.7340 with a SD of 0.0170, and a median of 0.7349 with 0.6952 as the minimum and 0.7721 as the maximum.
4. Finally, the fat/lean ratio given by the device differs greatly from the values obtained when the value for fat mass and that for lean mass are divided. In this case, percentual variations go from −15.7% to 13.0% with a mean of −6.5 (SD ± 4.7%), when they are lower, and 5.7% (SD ± 3.6%) when they are higher.

## Conclusions

In the authors’ opinion, firstly, the results of this study, although with only a relatively small convenience sample, support the idea that not only microbiota modulation should be added to the common and traditional triad of diet, physical exercise and motivation, in the prevention and treatment of overweight, at least in young adult women, but, also, that the fourth element of the proposed tetrad can be achieved by modulation of the IMB, which includes a colon cleansing protocol and can well be carried out at home, as it uses neither sophisticated equipment nor costly or exotic products. Products used in it are widely available worldwide, and practically none of them can be considered as a medicament. As it is well accepted that most, if not all, CNCDs have common causes and, to a good extent, also a common pathway, the tetrad could, eventually, be useful for the treatment of many or most of them, especially in their early stages or, even better, as a good option for their prevention. This study can easily be replicated anywhere, but, of course, it would be advisable, not only, to treat a much larger sample, with a longer follow up, but, also, to perform a proper and thorough bioinformatics analysis of the IMB of the participants, to better explore possible mechanisms for the effects that the protocol seems to produce.

Secondly, BIA seems to be able to mirror and reflect the physiological changes produced by the treatment, especially in relation, not only with body composition, but, also, in relation to metainflammation, which can be considered a hallmark of overweight and all chronic diseases. Nevertheless, it is necessary to mention that the data given for what we call here BIA-2 variables, must be taken cautiously, as there are many assumptions, taken for granted, which are not necessarily very accurate.

## Supplementary information

**Table SI-1:** Complete list of variables included in the study (*n*=44) with units, type, subtype and medians. In the LG, a single measurement was carried out, while, in the OG, measurements were taken at four different times (T1-T4, see **Figure 1** in the main text). Data of the two OG subgroups (COG and EOG) were pulled together, and pre and post values were considered as follows: pre values, for the EOG, were those taken at week 1 (T1) and, for the COG group, those taken at week 8 (T3); post values for the EOG were those taken at week 3 (T2), and, for the COG, those taken at week 10 (T4). The list is ordered by the last column, which shows the relative change (as %) of the median value of each variable in the week following the treatment (post) compared to those taken in the previous week (pre).

**No.**	**Name**	**Units**	**Type**	**Sub-type**	**LG (n=8)**	**Treated OG (n=11)**		**Post - pre-**	***P-value***	**Significance**	**Medians normalised against LG**				
						**Pre-**	**Post-**				**LG**	**pre**	**post**	**Post - pre**	**%**
**1**	C-reactive protein	mg/l	BIA-0	+	0.2	4.2	0.5	−3.7	4.3E-06	Yes	100.0	2100.0	260.0	−1840.0	−87.6
**2**	Triglycerides	mg/dl	BIA-0	+	82.0	177.0	79.0	−98.0	9.8E-04	Yes	100.0	215.9	96.3	−119.5	−55.4
**3**	Thyroid stimulating hormone	uIU/ml	BIA-0	+	1.6	3.2	1.5	−1.8	4.9E-07	Yes	100.0	206.5	93.5	−112.9	−54.7
**4**	Very-low density lipoprotein	mg/dl	BIA-0	+	23.1	34.6	20.8	−13.8	2.2E-06	Yes	100.0	149.8	90.0	−59.7	−39.9
**5**	Total cholesterol/high density lipoprotein ratio	------	BIA-0	+	2.2	3.6	2.6	−1.0	4.2E-07	Yes	100.0	163.2	117.3	−45.9	−28.1
**6**	Low-density lipoprotein	mg/dl	BIA-0	+	43.5	88.6	64.4	−24.2	1.1E-05	Yes	100.0	203.7	148.0	−55.6	−27.3
**7**	Tecumseh test heart rate	#/min	BIA-0	+	85.0	108.0	86.0	−22.0	1.8E-07	Yes	100.0	127.1	101.2	−25.9	−20.4
**8**	Total cholesterol	mg/dl	BIA-0	+	121.7	163.7	134.0	−29.7	9.8E-04	Yes	100.0	134.5	110.1	−24.4	−18.1
**9**	Resting heart rate	#/min	BIA-0	+	70.0	84.0	72.0	−12.0	9.0E-05	Yes	100.0	120.0	102.9	−17.1	−14.3
**10**	Fat/lean ratio	------	BIA-2	+	0.4	0.6	0.5	−0.1	3.0E-03	Yes	100.0	153.7	132.4	−21.3	−13.9
**11**	Fasting blood glucose	mg/dl	BIA-0	+	77.0	93.0	81.0	−12.0	5.9E-05	Yes	100.0	120.8	105.2	−15.6	−12.9
**12**	FMI (fat mass index)	%	BIA-2	+	5.7	9.7	8.5	−1.2	2.1E-04	Yes	100.0	168.8	147.9	−20.9	−12.4
**13**	Waist perimeter	cm	BIA-0	+	75.2	93.0	84.2	−8.8	1.1E-08	Yes	100.0	123.7	112.0	−11.7	−9.5
**14**	Systolic blood pressure	mmHg	BIA-0	+	109.0	121.0	110.0	−11.0	5.1E-05	Yes	100.0	111.0	100.9	−10.1	−9.1
**15**	Diastolic blood pressure	mmHg	BIA-0	+	70.0	78.0	71.0	−7.0	3.2E-07	Yes	100.0	111.4	101.4	−10.0	−9.0
**16**	Waist perimeter/height ratio	------	BIA-0	+	0.5	0.6	0.5	0.0	3.2E-08	Yes	100.0	118.8	108.3	−10.4	−8.8
**17**	Percentage body fat mass	%	BIA-2	+	23.6	34.5	31.9	−2.6	2.1E-10	Yes	100.0	146.2	135.2	−11.0	−7.5
**18**	Z5 (electrical impedance at 5 kHz)	Ω	BIA-1	+	794.0	711.0	666.0	−45.0	1.2E-01	No	100.0	89.5	83.9	−5.7	−6.3
**19**	Z20 (electrical impedance at 20 kHz)	Ω	BIA-1	+	769.0	681.0	639.0	−42.0	1.1E-01	No	100.0	88.6	83.1	−5.5	−6.2
**20**	Body mass index	kg/m^2^	BIA-0	+	21.3	28.0	26.3	−1.7	9.8E-04	Yes	100.0	131.5	123.7	−7.9	−6.0
**21**	Z50 (electrical impedance at 50 kHz)	Ω	BIA-1	+	711.5	611.0	577.0	−34.0	9.7E-02	No	100.0	85.9	81.1	−4.8	−5.6
**22**	R50 (electrical resistance at 50 kHz)	Ω	BIA-1	+	707.4	606.2	572.7	−33.5	9.5E-02	No	100.0	85.7	81.0	−4.7	−5.5
**23**	Z100 (electrical impedance at 100 kHz)	Ω	BIA-1	+	692.0	593.0	561.0	−32.0	9.5E-02	No	100.0	85.7	81.1	−4.6	−5.4
**24**	Z200 (electrical impedance at 200 kHz)	Ω	BIA-1	+	648.5	554.0	525.0	−29.0	9.2E-02	No	100.0	85.4	81.0	−4.5	−5.2
**25**	Weight	kg	BIA-0	+	52.1	69.9	66.5	−3.4	4.3E-11	Yes	100.0	134.3	127.8	−6.5	−4.9
**26**	Impedance ratio (IR) times 10	------	BIA-1	+	7.6	8.0	7.8	−0.1	1.7E-04	Yes	100.0	104.1	102.3	−1.8	−1.8
	Age	years	BIA-0		22.0	22.6	22.6	0.0	6.3E-02	No	100.0	103.0	103.0	0.0	0.0
	Height	cm	BIA-0		155.4	160.2	160.5	0.3	9.8E-04	Yes	100.0	103.1	103.3	0.2	0.2
**1**	Fat-free hydration	%	BIA-1	-	69.6	67.3	67.8	0.5	3.6E-01	No	100.0	96.8	97.5	0.7	0.7
**2**	Muscle mass index	------	BIA-1	-	1.0	1.2	1.2	0.0	5.7E-01	No	100.0	116.9	119.9	3.0	2.6
**3**	Total body water	%	BIA-2	-	52.4	44.2	46.0	1.8	7.1E-03	Yes	100.0	84.4	87.8	3.4	4.0
**4**	Phase angle	°	BIA-1	-	7.6	6.8	7.1	0.3	1.3E-04	Yes	100.0	89.9	93.8	3.9	4.4
**5**	Lean mass	%	BIA-2	-	70.1	59.9	62.8	2.9	1.1E-02	Yes	100.0	85.5	89.6	4.1	4.8
**6**	Active cell mass	%	BIA-2	-	44.8	38.2	40.6	2.4	8.4E-03	Yes	100.0	85.3	90.6	5.3	6.2
**7**	Extracellular water	%	BIA-2	-	19.0	15.9	17.0	1.1	1.3E-02	Yes	100.0	83.3	89.1	5.8	7.0
**8**	Intracellular water	%	BIA-2	-	29.2	23.7	25.5	1.8	1.6E-03	Yes	100.0	81.2	87.3	6.1	7.5
**9**	Skeletal muscle mass	%	BIA-2	-	36.9	32.4	35.0	2.6	4.5E-03	Yes	100.0	87.8	95.0	7.2	8.2
**10**	Metabolic protein mass	%	BIA-2	-	14.4	13.0	14.1	1.1	2.5E-01	No	100.0	90.3	98.2	7.9	8.8
**11**	High-density lipoprotein	mg/dl	BIA-0	-	54.5	45.0	52.0	7.0	1.4E-07	Yes	100.0	82.6	95.4	12.8	15.6
**12**	Xc50 (capacitive reactance at 50 kHz)	Ω	BIA-1	-	77.6	71.1	82.2	11.1	1.0E-01	No	100.0	91.6	105.9	14.3	15.7
**13**	Modified Wells sit-and-reach test	cm	BIA-0	-	41.1	28.0	33.0	5.0	7.1E-08	Yes	100.0	68.1	80.3	12.2	17.9
**14**	Maximal hand grip strength	kg	BIA-0	-	33.1	25.8	32.7	6.9	1.4E-06	Yes	100.0	77.9	98.8	20.8	26.7
**15**	Firmicutes/bacteroidota ratio	------	BIA-0	-	1.9	0.9	1.6	0.6	2.1E-01	No	100.0	51.2	84.3	33.2	64.9
**16**	Relative prevalence of *Akkermansia muciniphila*	%	BIA-0	-	1.1	0.1	6.1	6.0	1.5E-02	Yes	100.0	9.0	568.6	559.6	6197.8

**Table SI-2.** Main products consumed during the 6-day treatment, indicating daily intake, caloric content, and macronutrient composition (protein, carbohydrates, and fat).

**Product**	**Daily intake**	**Units**	**Calories**	**Protein (g)**	**Carbohydrates (g)**	**Fat (g)**
Almond milk	250	ml	37.5	1.0	2.5	0.0
Apple juice	415	ml	207.5	0.0	50.0	5.0
Apple vinegar	100	ml	24.0	0.0	0.0	0.0
Avocado	80	g	143.0	1.2	8.0	7.3
Bee honey	75	ml	249.0	0.4	61.8	0.1
Broth made of ginger, carrot, celery, mushroom, parsley, spinach, and pumpkin.	250	ml	52.0	1.4	9.2	0.4
Coca tea	100	ml	0.0	0.0	0.0	0.4
Cold pressed 100% vegetable juice made of peppermint, apple, cucumber & wheatgrass.	500	ml	92.0	1.0	21.1	11.0
Granulated kola (multivitamin supplement)	15	g	52.5	0.0	11.6	1.2
Greek yogurt	100	g	66.6	7.3	0.6	8.6
Kefir/kombucha	20	ml	12.8	0.7	0.7	0.0
Liquid chlorophyll	30	ml	0.0	0.0	0.0	0.6
Liquid chocolate	200	ml	93.0	0.7	6.3	1.0
Montmorillonite (clay)	15	g	0.0	0.0	0.0	0.0
Papaya	160	g	64.0	0.8	13.1	0.0
Psyllium	15	g	0.0	0.0	0.0	0.0
Quinoa powder	15	g	56.0	2.1	22.5	0.0
**Total**			**1149.9**	**16.6**	**207.4**	**35.6**

**Table SI-3.** List of abbreviations and symbols.

“−”	Subtype of variables whose increase can be considered as beneficial
“+”	Subtype of variables whose decrease can be considered as beneficial
%AM	Relative prevalence of *Akkermansia muciniphila* bacteria
%BF	Percentage of body fat
BIA	Bioelectrical impedance analysis
BIA-0	Type of variables not involving BIA values
BIA-1	Type of electrical measured/calculated BIA variables
BIA-2	Type of variables combining BIA-0 an BIA-1 values
BMI	Body mass index
CBCS	Bioethical Committee of Health Sciences (Comité de Bioética de Ciencias para la Salud, in Spanish)
CI95%	Confidence interval at 95% level
CNCDs	Chronic non communicable diseases
COG	Control (sub)group of overweight volunteers
CRP	C-reactive protein
EOG	Experimental (sub)group of overweight volunteers
F/BR	*Firmicutes*/*bacteroidota* ratio
HDL	High density lipoprotein or “good” cholesterol
IMB	Intestinal microbiota
IR	Impedance ratio (Z200/Z5)
LDL	Low density lipoprotein or "bad" cholesterol
LG	Group of lean volunteers
OG	Group of overweight volunteers
PhA	Phase angle at 50 kHz
R50	Electrical resistance (or real part of the impedance, Z’) at 50 kHz in ohm (Ω)
SD	Standard deviation
SI	Supplementary information
T1	Time of first measurements (week 1)
T2	Time of second measurements (week 4)
T3	Time of third measurements (week 8)
T4	Time of fourth measurements (week 10)
TG	Triglycerides
Xc50	Electrical reactance (or imaginary part of Z, Z”) at 50 kHz in Ω (ohm)
